# Plasma Metabolite Profiles of Children with Autism Spectrum Disorder

**DOI:** 10.3390/metabo15120780

**Published:** 2025-12-04

**Authors:** Benjamin H. Mullin, Madeleine Stuckey, Suzanne J. Brown, Shelby Mullin, Purdey J. Campbell, Frank Dudbridge, Cristina Menni, John P. Walsh, Andrew J. O. Whitehouse, Scott G. Wilson

**Affiliations:** 1 Department of Endocrinology & Diabetes, Sir Charles Gairdner Hospital, Nedlands, WA 6009, Australia; 2 Medical School, University of Western Australia, Crawley, WA 6009, Australia; 3 Department of Population Health Sciences, University of Leicester, Leicester LE1 7RH, UK; 4 Department of Twin Research and Genetic Epidemiology, King’s College London, London SE1 7EH, UK; 5 Department of Pathophysiology and Transplantation, Università Degli Studi di Milano, Via Francesco Sforza, 35, 20122 Milan, Italy; 6 Angelo Bianchi Bonomi Hemophilia and Thrombosis Center, Fondazione IRCCS Cà Granda Ospedale Maggiore Policlinico, 20122 Milan, Italy; 7 The Kids Research Institute Australia, The University of Western Australia, Nedlands, WA 6009, Australia; 8 Cooperative Research Centre for Living with Autism (Autism CRC), Long Pocket, QLD 4850, Australia; 9 School of Biomedical Sciences, University of Western Australia, Crawley, WA 6009, Australia

**Keywords:** autism, metabolomics, autism spectrum disorder, restricted and repetitive behaviours and interests

## Abstract

**Background/Objectives:** Autism spectrum disorder (ASD), a neurodevelopmental condition characterised by social and communication differences, is complex and aetiologically heterogeneous. Untargeted metabolomics is emerging as a tool in screening for biochemical abnormalities. This research was conducted using the Australian Autism Biobank resource and involved analysis of plasma metabolites to characterise metabolite differences between autistic children and controls. **Methods:** We sought to identify molecular signatures in the plasma of study subjects using mass-spectrometry methods. We included 955 untargeted plasma metabolites from autistic children (*n* = 491; 2–18 years; 78% male) and control subjects (n = 97; 2–17 years of age; 51% male). Statistical analyses were performed using questionnaire data for both groups, including standardised scores from the Autism Diagnostic Observation Schedule—Second Edition (ADOS-2), which measures the severity of autism-related behaviours. We also evaluated intellectual disability by examining the relationships between metabolites and clinical phenotypes. **Results:** After controlling the false discovery rate at 5%, we identified significant negative associations between the uncharacterised metabolites X-21383 and X-24970 and ASD status (*p* = 1.85 × 10^−6^ and *p* = 1.92 × 10^−5^ respectively). X-21383 was also found to be significantly reduced in autistic children with coexisting intellectual disability when compared with controls (*p* = 6.06 × 10^−6^). No significant associations were identified between the metabolite data and ADOS-2 scores. However, greater levels of X-16938, N1-methyladenosine, and 2-oxoarginine were found to be suggestively associated with higher ADOS-2 scores (*p* = 2.95 × 10^−4^–9.6 × 10^−5^). **Conclusion:** This metabolomics study in the Australian Autism Biobank has identified several novel metabolites associated with core autism diagnostic behaviours.

## 1. Introduction

Autism spectrum disorder (ASD) is a neurodevelopmental condition characterised by social and communication differences, restricted and repetitive behaviours, and differences in sensory sensitivity [1]. Currently, ASD is diagnosed by observational evaluation of behavioural characteristics and developmental progression. However, identification of unique biochemical or metabolomic characteristics could aid in early and efficient diagnosis and could potentially lead to the development of new therapies that can support quality of life.

Metabolic differences have previously been reported in some autistic individuals [2]; however, many of the existing studies have measured only a subset of metabolites of known chemical identity. In addition, reported outcomes have often not been reproduced between studies. Large-scale untargeted metabolomics is emerging as a tool for efficient detection of unique biochemical and metabolomic characteristics and biochemical abnormalities, including in autistic people [3]. These studies characterise a large number of small molecules that form part of the metabolome, typically through analysis of tissue, blood (plasma or serum), or stool samples. Metabolites are postulated to play foundational roles as effectors and biomarkers in several complex neurological conditions, and there is evidence to suggest that the metabolome is heritable to differing degrees across metabolite classes [4]. Therefore, identified biomarkers or metabolic signatures (groups of related biomarkers) offer a potentially attractive adjunct to aid diagnosis and treatment of many diseases.

In this study, we hypothesised that a proportion of autistic individuals exhibit a distinct metabolite signature that provides insight into the aetiology of autism-related behaviour. We investigated whether specific metabolites and metabolic pathways could discriminate between autistic children and controls, as well as between autistic children with and without intellectual disability and controls. Additionally, we examined whether metabolite levels were associated with measures of severity of autistic diagnostic behaviours. Our aim was to refine and broaden the understanding of circulating metabolite profiles and their association with characteristic features of ASD.

## 2. Materials and Methods

### 2.1. Data Collection

Biological samples of plasma extracted from whole blood were available for this study, having been collected previously by the Australian Autism Biobank (AAB) from four autism research clinics around Australia (Perth, Sydney, Melbourne, and Brisbane) [5]. Participants included children aged between 2 and 18 years old. The ASD case group consisted of 491 children with a clinically confirmed ASD diagnosis according to the Diagnostic and Statistical Manual of Mental Disorders, Fourth and Fifth edition (DSM-IV, DSM-V) criteria. The control group comprised 97 children with no known developmental differences, including neurotypical siblings of the ASD cases and unrelated children with no family history of ASD.

### 2.2. Cognitive and Behavioural Assessments

The autistic children were assessed using the Autism Diagnostic Observation Schedule—Second Edition (ADOS-2) [6], which is a semi-structured assessment that assesses communicative and social behaviours relevant to the diagnosis of autism using simple activities and questions. The activities can be tailored to the individual’s level of verbal language using a range of modules (1, 2, 3, or 4). The ADOS-2 calibrated severity score (ranging from 1 to 10 points) was designed to enable comparisons across different developmentally staged ADOS-2 modules [7]. Higher scores indicate a greater manifestation of autism-related behavioural characteristics.

Cognitive abilities were assessed using either the Mullen Scales of Early Learning (MSEL) or the Wechsler Intelligence Scale for Children—Fourth Edition (WISC-IV), depending on the participant’s age. For children aged 2 to 6 years, the MSEL was used, which is a standardised developmental assessment tool that involves interactive, play-based tasks [8]. Four domains were evaluated: (i) fine motor, (ii) visual reception, (iii) expressive language, and (iv) receptive language. A non-verbal IQ (NVIQ) was calculated based on a method validated by Bishop et al. [9]: age-equivalent scores from the fine motor and visual reception domains were averaged, divided by the child’s chronological age, and multiplied by 100. Intellectual disability was defined as an NVIQ < 70. For children older than 6 years, the WISC-IV [10] was used. This includes ten structured tasks that assess cognitive abilities in four areas: (i) verbal comprehension, (ii) perceptual reasoning, (iii) working memory, and (iv) processing speed. Intellectual disability was defined as a full-scale IQ < 70.

### 2.3. Sample Preparation

Plasma samples were prepared using the automated MicroLab STAR^®^ system (Hamilton Company, Reno, NV, USA). Briefly, recovery standards were added to each sample before proteins were precipitated with methanol under vigorous shaking for 2 min (Glen Mills GenoGrinder 2000, Clifton, NJ, USA) followed by centrifugation. The resulting extract was split into five fractions: two for analysis by separate reverse-phase (RP) ultrahigh-performance liquid chromatography–tandem mass spectroscopy (UPLC-MS/MS) methods with positive-ion-mode electrospray ionisation (ESI), one for analysis by RP/UPLC-MS/MS with negative-ion-mode ESI, one for analysis by HILIC/UPLC-MS/MS with negative-ion-mode ESI, and the final fraction reserved for backup. Organic solvent was removed from the samples by placing them briefly on a TurboVap^®^ (Zymark, Hopkinton, MA, USA) before they were stored under nitrogen overnight until analysis.

### 2.4. Quality Control

A pooled matrix sample, made up of a small volume of each experimental sample or well-characterised human plasma, was included throughout the sample set as a technical replicate. Ultra-pure water samples were included as process blanks, and every analysed sample was spiked with QC standards carefully selected so as not to interfere with the measurement of endogenous compounds. These allowed for monitoring of instrument performance and aided chromatographic alignment. The median relative standard deviation (RSD) was calculated for the standards that were added to each sample to assess instrument variability. The median RSD was calculated for all endogenous metabolites present in the pooled matrix samples to determine overall process variability. QC samples were spaced evenly among the injections, with experimental samples randomised across the platform run.

### 2.5. Metabolite Profiling

Global metabolite profiling was performed for all cases and control children (total 588 plasma samples) using non-targeted mass spectrometry (MS) on available plasma samples by Metabolon, Inc. (Durham, NC, USA), as described previously [11]. Briefly, UPLC-MS/MS was conducted using a Waters ACQUITY UPLC system coupled to a Thermo Scientific Q-Exactive high-resolution/accurate mass spectrometer equipped with a heated electrospray ionisation (HESI-II) source [12] and an Orbitrap mass analyser operated at a 35,000 mass resolution. Sample extracts were reconstituted using solvents containing fixed concentrations of a series of standards used to ensure injection and chromatographic consistency. Two aliquots of each sample were analysed using acidic positive-ion conditions, one chromatographically optimised for more hydrophilic compounds and the other for more hydrophobic compounds. The aliquot optimised for hydrophilic compounds was gradient-eluted from a C18 column (UPLC BEH C18 Column, 130Å, 1.7 μm, 2.1 mm × 100 mm) using water and methanol containing 0.05% perfluoropentanoic acid (PFPA) and 0.1% formic acid (FA). The aliquot optimised for hydrophobic compounds was gradient-eluted from the same C18 column using methanol, acetonitrile, water, 0.05% PFPA, and 0.01% FA and was operated at an overall higher organic content. One aliquot of each sample was analysed using basic negative-ion-optimised conditions with a separate dedicated C18 column. The basic extracts were gradient-eluted from the column using methanol, water, and 6.5 mM ammonium bicarbonate at pH 8. The fourth aliquot of each sample was analysed using negative ionisation following elution from a HILIC column (Waters UPLC BEH Amide 2.1 mm × 150 mm, 1.7 µm) using a gradient consisting of water and acetonitrile with 10mM ammonium formate, pH 10.8. MS analysis alternated between MS and data-dependent MS^n^ scans using dynamic exclusion, with the scan range varied between methods but covering 70–1000 *m*/*z*.

The area-under-the-curve method was used to quantify the metabolites. The raw area count for each metabolite in each sample was standardised to reduce variation originating from differences in daily instrument tuning. To achieve this, the median values for each day of the run were adjusted to 1.0, with each data point normalised proportionately. This allowed for comparison of metabolites with significantly different raw peak areas on a uniform scale while maintaining biologically relevant sample-to-sample variation. Compounds were identified by comparison to Metabolon’s chemical reference library of authenticated standards or recurrent unknown entities, analysed under identical instrumental conditions as the samples. Three criteria were used to identify compounds: a retention index within a narrow window of the proposed metabolite, a mass match within ±10 ppm, and comparison of the MS/MS forward and reverse scores (i.e., comparison of the ions present in the sample spectrum to those present in the library spectrum). Metabolite identification confidence was assigned using the standard five-level MSI/Schymanski framework, mapping Metabolon annotations such that named biochemicals correspond to Levels 1–2 and unnamed X-features to Level 5, as detailed in the Supplementary Materials. A total of 1291 metabolites were detected; of these, 1041 were of known and 250 were of unknown chemical identity. Batch normalisation was performed on the metabolite data, and any metabolites demonstrating >20% missingness were removed from the dataset. Values missing due to low metabolite concentration were imputed using the day minimum value for that metabolite before the dataset was subjected to inverse normalisation.

### 2.6. Statistical Analysis

We first explored the relationships between metabolites and ASD traits using a linear mixed-effects model accounting for age, BMI, sex, technical covariates (sample box and sample row), and family relatedness between participants. We compared (i) all ASD cases vs. controls, (ii) ASD cases with coexisting intellectual disability vs. controls, and (iii) ASD cases with no intellectual disability vs. controls. In the group with ASD, the same modelling approach was used to identify associations between compounds and ADOS-2 score. Correction for multiple testing was performed using the Benjamini–Hochberg procedure [13] with a false discovery rate (FDR) of 5% (corrected Q ≤ 0.05 considered significant). All analyses were performed using the R (R 4.4.3) statistical computing environment [14].

## 3. Results

### 3.1. Descriptive Statistics

Descriptive statistics for the study cohort are presented in Table 1. As expected, given the known sex imbalance in ASD diagnosis, there was a higher proportion of males in the ASD group (78%) than in the controls (51%). The ASD group were also slightly older and heavier than the control group. Metabolomics analysis of the plasma samples yielded data for 1291 metabolites, of which 40 (3%) corresponded to known drugs, most of which recorded non-zero quantifications in only one or two subjects. Of the remaining 1251 metabolites, 296 had missingness >20% so were removed, leaving 955 metabolites for analysis for association with ASD (Supplementary Table S1).

### 3.2. Associations Identified Between Metabolites and ASD

**Figure 1:**
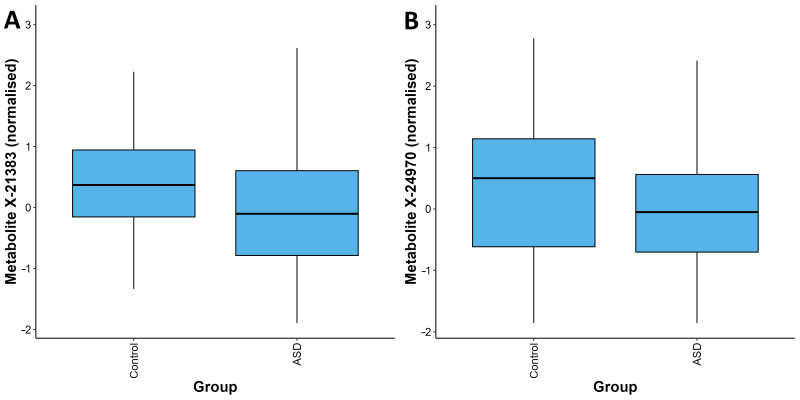
Distribution of quantifications for the metabolites X-21383 (**A**) and X-24970 (**B**) in the ASD and control groups. For each metabolite, the values in the ASD group are significantly lower than those in the control group (*p* = 1.85 × 10^−6^ and *p* = 1.92 × 10^−5^ respectively). The box plots depict the minimum, first quartile, median, third quartile, and maximum values for each group.

**Figure 2:**
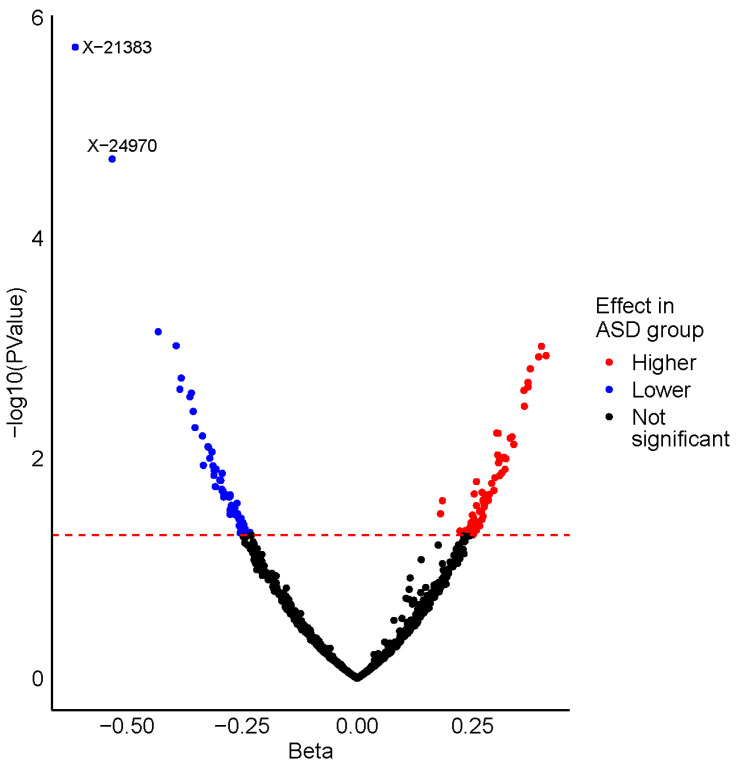
Volcano plot displaying the results from the ASD vs. control analysis. Metabolites are plotted according to their beta value (*x*-axis) and -log_10_ (*p*-value) (*y*-axis), with the horizontal dashed red line representing the nominal significance threshold (*p* = 0.05). Nominally significant metabolites identified with a higher level in the ASD group are coloured red, whereas those with a lower level in the ASD group are coloured blue. The two metabolites that are significantly associated with ASD status after correction for multiple testing are labelled.

**Figure 3:**
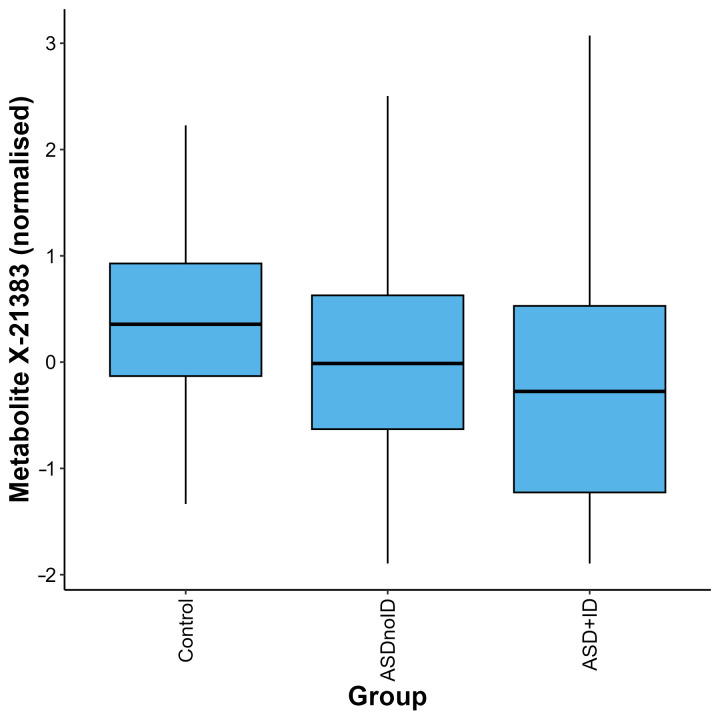
Distribution of quantifications for the metabolite X-21383 in the control, ASD without intellectual disability (ASDnoID), and ASD with intellectual disability (ASD + ID) groups, defined using the MSEL/NVIQ or WISC criteria. The metabolite X-21383 was found to be present at lower levels in the ASD + ID and ASDnoID groups when compared with the controls (*p* = 6.06 × 10^−6^ and 2.32 × 10^−4^, respectively). The box plot depicts the minimum, first quartile, median, third quartile, and maximum values for each group.

After adjustment for relevant covariates, we identified 126 metabolites as nominally associated with ASD status (Supplementary Table S2). Two of these metabolites remained significantly associated with ASD status after correction for multiple testing, X-21383 and X-24970 (*p* = 1.85 × 10^−6^ and *p* =1.92 × 10^−5^, respectively, Table 2, Figure 1A,B and Figure 2, Supplementary Table S3). Autistic children were further stratified into two groups, those with an intellectual disability and those without. When the complete metabolite profiles of autistic children with intellectual disability were compared to those of the controls, the metabolite X-21383 was found to be present at significantly lower levels in the cases (*p =* 6.06 × 10^−6^, Table 2 and Figure 3, Supplementary Table S3). No significant associations were identified when autistic children without intellectual disability were compared with controls after correction for multiple testing. However, both X-21383 (Figure 3, Supplementary Table S3) and X-24970 were negatively associated at nominal level (*p* = 2.32 × 10^−4^ and 9.05 × 10^−4^, respectively).

### 3.3. Analysis of Metabolome with ADOS-2 Scores

Within the ASD case population, we further tested the association between circulating metabolites and ADOS-2 scores. Though we found no significant associations after adjusting for multiple testing, suggestive positive associations (Q ≤ 0.1) were observed for the metabolites X-16938, N1-methyladenosine, and 2-oxoarginine (*p* = 2.95 × 10^−4^–9.6 × 10^−5^, Table 3).

## 4. Discussion

In this study, we identified significant associations between ASD status and two metabolites of unknown chemical identity, X-21383 and X-24970. Importantly, X-21383 showed strong associations across all ASD cases, including those with coexisting intellectual disability. Autistic children presented with lower levels of X-21383, with a subset of these individuals (6.9%) recording quantifications below the assay’s minimum detection threshold. Previous studies have linked X-21383 to a BMI-predictive omics model, where it was reported to be negatively associated with BMI [15]; it is also thought to be correlated with dietary consumption of roti and deep-fried foods [16]. These findings may reflect distinct dietary preferences commonly reported in autistic children [17].

We found circulating levels of N1-methyladenosine, X-16938, and 2-oxoarginine to be positively associated with higher ADOS-2 scores (at a nominal level). N1-methyladenosine is an adenosine moiety with a methylated N1-position that has an important role in the biogenesis of RNA molecules. Its presence has been found to influence the secondary structure of eukaryotic mRNA molecules, enhancing their translation efficiency and subsequent protein production [18]. Qi and coworkers [19] found that N1-methyladenosine modification of mRNA is abundant in mouse cortical neurons, and its levels were significantly increased after oxygen glucose deprivation/reoxygenation induction, suggesting a potential role in neurological damage. They also found that differential N1-methyladenosine peaks are enriched in pathways linked with neurological diseases and oxidative stress damage. 2-oxoarginine is a guanidino metabolite of arginine catabolism. Elevated levels of 2-oxoarginine are seen in patients with argininemia [20], an autosomal recessive disorder caused by a deficiency in the arginase enzyme. The accumulation of 2-oxoarginine in these individuals has been suggested to contribute to the central nervous system damage associated with the disorder [21]. Further studies are required to establish whether N1-methyladenosine and 2-oxoarginine have a role in the pathophysiology of ASD.

It should be acknowledged that while this is among the largest metabolomics studies of ASD conducted to date, the sample size is still relatively small, particularly for the ASD with/without coexisting intellectual disability subgroup analyses. However, it should be noted that significant discoveries in this field have been made with smaller sample sizes than those used in this study [3]. Other potential limitations include reverse causation and unobserved confounding due to the observational nature of the study. The significant associations seen for metabolites that have been previously linked with dietary habits could potentially be a product of this, considering that autistic children often exhibit food aversions leading to reduced dietary diversity [22].

In conclusion, we have identified significant associations between the metabolites X-21383 and X-24970 and ASD status, both of which were present at a lower level in autistic children compared to controls. X-21383 was also significantly reduced in autistic children with coexisting intellectual disability. While no significant associations were identified between the metabolome and ADOS-2 scores, suggestive associations were seen between greater levels of the adenosine moiety N1-methyladenosine and the guanidino compound 2-oxoarginine and higher ADOS-2 scores, possibly reflecting a role for these metabolites in neurological damage. Collectively, these findings provide additional support for a potential link between the metabolome and autism.

## Figures and Tables

**Table 1:** Demographic data for the study cohort.

Variable	ASD			Control			p *
	Males n = 384	Females n = 107	Combined n = 491	Males n = 49	Females n = 48	Combined n = 97	
Age	6.9 (3.8)	7.6 (4.0)	7.1 (3.8)	5.8 (2.8)	6.7 (3.8)	6.2 (3.3)	0.05
Height	124 (25.8)	128 (22.9)	125 (25.2)	120 (19.3)	122 (23.6)	121 (21.5)	0.19
Weight	30.9 (20.9)	31.4 (16.8)	31.0 (20.1)	25.3 (10.6)	27.8 (14.9)	26.7 (12.9)	0.04
BMI	17.5 (3.9)	18.1 (3.9)	17.6 (3.9)	16.9 (2.6)	17.5 (3.2)	17.2 (2.9)	0.34
MSEL	69.1 (22.2)	78.0 (27.3)	70.9 (23.6)	101 (15.8)	105 (18.1)	102 (16.8)	2.71 × 10^−16^
WISC-IV	86.9 (21.0)	87.4 (22.8)	87.0 (21.3)	98.2 (12.4)	107 (11.7)	103 (12.6)	1.92 × 10^−6^
ADOS-2	6.7 (1.8)	6.6 (2.0)	6.7 (1.9)	-	-	-	NA
ID	111 (34%)	29 (32%)	140 (33%)	0	0	0	NA

Values shown are mean (SD) or count (%), where the percentage is based on those for whom data are available. Controls did not undergo ADOS-2 assessment; ADOS-2 scores were available for 477 (97%) of children on the spectrum. BMI: body mass index, MSEL: Mullen Scales of Early Learning, WISC-IV: Wechsler Intelligence Scale for Children—Fourth Edition, ADOS-2: Autism Diagnostic Observation Schedule—Second Edition, ID: intellectual disability defined using MSEL/NVIQ or WISC. * *p* values were derived from comparing the ‘Combined’ groups for ASD and controls.

**Table 2:** Metabolites identified as significantly associated with ASD traits.

Analysis	Metabolite	Beta	SE	*p*	*Q*
ASD vs. control	X-21383	−0.61	0.13	1.85 × 10^−6^	0.002
	X-24970	−0.53	0.12	1.92 × 10^−5^	0.009
ASD + ID vs. control	X-21383	−0.72	0.14	6.06 × 10^−6^	0.006

Statistical analyses were adjusted for age, sex, BMI, and relevant technical covariates (sample box and sample row). *Q* values were corrected for multiple testing using the Benjamini–Hochberg procedure. ASD + ID: ASD cases with coexisting intellectual disability, SE: standard error. Beta values represent the change in mean transformed metabolite value between ASD and controls.

**Table 3:** Metabolites identified as suggestively associated with ADOS-2 score.

Analysis	Metabolite	Beta	SE	*p*	*Q*
ADOS-2 score	X-16938	0.11	0.02	9.6 × 10^−5^	0.09
	N1-methyladenosine	0.10	0.03	2.14 × 10^−4^	0.09
	2-oxoarginine *	0.10	0.03	2.95 × 10^−4^	0.09

Statistical analyses were adjusted for age, sex, BMI, and relevant technical covariates (sample box and sample row). *Q* values were corrected for multiple testing using the Benjamini–Hochberg procedure. * Metabolite identity not definitively confirmed based on a standard.

## Data Availability

The metabolomics data used in this study will be made available by the authors upon request to the Autism CRC/Australian Autism Biobank.

## Supplementary Materials

The following supporting information can be downloaded at: https://www.mdpi.com/article/10.3390/metabo15120780/s1, Table S1: Complete list of the 955 metabolites included in the analysis and their annotation levels; Table S2: Results for the 126 nominally significant metabolites from the ASD vs control analysis; Table S3: Normalised metabolite distributions across groups for X-21383 and X-24970. Supplementary File: Metabolite identification confidence levels [23,24].

## Abbreviations

The following abbreviations are used in this manuscript:

ASD	Autism spectrum disorder
ADOS-2	Autism Diagnostic Observation Schedule—Second Edition
AAB	Australian Autism Biobank
MSEL	Mullen Scales of Early Learning
WISC-IV	Wechsler Intelligence Scale for Children—Fourth Edition
NVIQ	Non-verbal IQ
MS	Mass spectrometry
BMI	Body mass index
FDR	False discovery rate
ID	Intellectual disability
