# Supplementary material for: Plasma Metabolite Profiles of Children with Autism Spectrum Disorder
Source: Metabolites. 2025 Dec 4;15(12):780. doi: 10.3390/metabo15120780 (PMC12734473; doi:10.3390/metabo15120780)
Supplement: Supplementary file 1 [file metabolites-15-00780-s001.zip › Supplementary_Methods_271125.pdf]

Supplementary Methods

Metabolite identification confidence levels

Metabolite identification was performed by Metabolon Inc. (Morrisville, NC, USA) using their established untargeted LC–MS/MS platform. Identification relies on accurate mass, retention time/index, and MS/MS spectral matching against Metabolon's proprietary library of authenticated standards and recurrent unknowns. To align with community standards [1], we classified all compounds according to the five-level identification confidence framework adapted from the Metabolomics Standards Initiative and Schymanski et al [2]:

* Level 1 – structure confirmed by comparison of retention time (tR), MS1 and MS2 spectra with an authentic standard;
* Level 2 – probable structure based on concordant tR, MS1 and MS2 spectra matched to a library entry (mass error <10 ppm, tR deviation <0.5 min, MS2 spectral similarity >0.5);
* Level 3 – putative identification based on tR and MS1 only;
* Level 4 – unequivocal molecular formula established from MS1;
* Level 5 – mass spectral feature with insufficient evidence to assign structure.

Metabolon's internal annotation flags were mapped onto these levels as follows: Biochemical Name (no symbol) corresponded to Level 1 (confirmed using an authentic standard). Biochemical Name* and Biochemical Name** entries, which represent high-confidence identifications lacking a standard, were assigned to Level 2. Compounds annotated as Biochemical Name (#) or [#], reflecting unresolved structural isomerism, were also assigned to Level 2 (probable structure with isomeric ambiguity). Features reported as UNNAMED (X-#####) lack a defined structure and were therefore assigned to Level 5. Because Metabolon does not provide per-metabolite tR deviations or MS2 similarity scores in client deliverables, finer subdivision of Levels was not possible.

References

1. Wang, Z.; Sun, Y.; Chen, T.; Jiang, L.; Shang, Y.; You, X.; Hu, F.; Yu, D.; Liu, X.; Wan, B.; et al. High-Coverage Profiling of Hydroxyl and Amino Compounds in Sauce-Flavor Baijiu Using Bromine Isotope Labeling and Ultra-High Performance Liquid Chromatography-High-Resolution Mass Spectrometry. *Metabolites* **2025**, *15*, doi:10.3390/metabo15070464

2. Schymanski, E.L.; Jeon, J.; Gulde, R.; Fenner, K.; Ruff, M.; Singer, H.P.; Hollender, J. Identifying small molecules via high resolution mass spectrometry: communicating confidence. *Environ Sci Technol* **2014**, *48*, 2097-2098, doi:10.1021/es5002105 [doi].
